# Mutations in the epidermal growth factor receptor gene are linked to smoking-independent, lung adenocarcinoma

**DOI:** 10.1038/sj.bjc.6602707

**Published:** 2005-07-26

**Authors:** M Sonobe, T Manabe, H Wada, F Tanaka

**Affiliations:** 1 Department of Thoracic Surgery, Kyoto University Hospital, Shogoin-Kawara-cho 54, Sakyo-ku, Kyoto 606-8507, Japan; 2 Laboratory of Anatomic Pathology, Kyoto University Hospital, Shogoin-Kawara-cho 54, Sakyo-ku, Kyoto 606-8507, Japan

**Keywords:** epidermal growth factor receptor, lung adenocarcinoma, smoking, p53, K-ras, single-strand conformation polymorphism

## Abstract

Epidermal growth factor receptor (EGFR) mutations are a potential predictor of the effectiveness of EGFR inhibitors for the treatment of lung cancer. Although EGFR mutations were reported to occur with high frequency in nonsmoking Japanese adenocarcinoma patients, the exact nature has not been fully elucidated. We examined EGFR gene mutations within exons 18–21 and their correlations to clinico-pathological factors and other genetic alterations in tumour specimens from 154 patients who underwent resection for lung cancer at Kyoto University Hospital. Epidermal growth factor receptor mutations were observed in 60 tumours (39.0%), all of which were adenocarcinoma. Among the patients with adenocarcinoma (*n*=108), EGFR mutations were more frequently observed in nonsmokers than former smokers or current smokers (83.0, 50.0, 15.2%, respectively), in women than men (76.3 *vs* 34.0%), in tumours with bronchio-alveolar component than those without bronchio-alveolar component (78.9 *vs* 42.9%), and in well or moderately differentiated tumours than poorly differentiated tumours (72.0, 64.4, 34.2%). No tumours with EGFR mutations had any K-ras codon 12 mutations, which were well-known smoking-related gene mutations. In conclusion, adenocarcinomas with EGFR mutation had a distinctive clinico-pathological feature unrelated to smoking. Epidermal growth factor receptor mutations may play a key role in the development of smoking-independent adenocarcinoma.

Lung cancer is the leading cause of cancer death in many industrialised countries. Cigarette smoking is the most important cause of lung cancer, and a number of smoking-related gene alternations have been identified that are responsible for the development of lung cancer, such as mutations in K-ras (Vineis and Caporaso, 1995; Shields, 2002). However, lung cancer also develops in nonsmokers, and 30–40% of the lung cancer patients in Japan have never smoked history are female, and their major histological tumour type is adenocarcinoma (Sobue *et al*, 1994; Akazawa *et al*, 2003). While several reports have shown that the adenocarcinomas that occurred in nonsmokers were distinct from those that developed in smokers in terms of their histological subclassification, prognosis, gene expression pattern, and gene alterations (Tsuchiya *et al*, 1988; Hashimoto *et al*, 2000; Ahrendt *et al*, 2001; Bhattacharjee *et al*, 2001; Koga *et al*, 2001; Noda *et al*, 2001; Vahakangas *et al*, 2001; Yang *et al*, 2002), few significant genetic alterations have been reported in adenocarcinomas that developed in nonsmokers.

Recent laboratory studies have shown that the epidermal growth factor receptor (EGFR, also known as ErbB1 or HER1) plays a critical role in the development and progression of a variety of malignant tumours by promoting cell growth, and by preventing apoptosis through regulation of downstream effectors such as mitogen-activated protein kinase, protein kinase B, and signal transducer and activator of transcription 3 (Klapper *et al*, 2000). It has been shown in clinical studies that EGFR is overexpressed in 40–80% of non-small-cell lung carcinomas (NSCLCs) (Bunn and Franklin, 2002), and in preneoplastic lesions (Flanklin *et al*, 2002). These findings suggest that EGFR might have the potential to be an important molecular target for the diagnosis and treatment of NSCLC. However, it is important to note that the exact role of measuring EGFR status in clinical setting remains unclear: EGFR expression status may not be useful as a prognostic tool (Meert *et al*, 2002), and may not predict responsiveness to treatment with gefitinib, a small-molecule EGFR tyrosine kinase inhibitor (Kris *et al*, 2003).

Lynch *et al* (2004) and Paez *et al* (2004) recently identified specific mutations in the tyrosine kinase domain of the EGFR gene within exons 18, 19, and 21 in most NSCLC patients who responded to gefitinib. Furthermore, Pao *et al* (2004) reported the presence of a point mutation in exon 20 of the EGFR gene in an adenocarcinoma patient who responded to erlotinib, another EGFR tyrosine kinase inhibitor. Lynch *et al* (2004) and Pao *et al* (2004) also showed that cancer cells transfected with EGFR gene mutations showed enhanced tyrosine kinase activity in response to binding of epidermal growth factor and increased sensitivity to gefitinib and erlotinib, suggesting that specific EGFR mutations may predict responsiveness to this type of treatment.

On the other hand, Paez *et al* (2004) and Pao *et al* (2004) reported that EGFR mutations were more frequent in female than in male patients and in adenocarcinomas than in tumours of other histological types. Moreover, reports objecting peoples in East Asia (Huang *et al*, 2004; Kosaka *et al*, 2004; Shigematsu *et al*, 2005) reported a half of adenocarcinomas in East Asia patients had EGFR mutations and the absence of smoking history, mainly seen in female patients, were closely linked to EGFR mutations. These reports are not only important in determining which patients should receive EGFR-targeted treatment (Arteaga, 2004; Dancey, 2004) but also indicate that EGFR mutations may play a causal role in the development of lung adenocarcinoma in nonsmokers.

To confirm the correlation of EGFR mutations with smoking, we conducted a detailed study of EGFR gene mutations in NSCLC patients who underwent tumour resection at a particular Japanese hospital. In the study, mutations of p53 gene and K-ras gene codon 12, and promoter hypermethylation status of p16, RASSF1A, and APC1A gene, were also examined because these gene alterations had substantial role in pathogenesis of NSCLC and whether or not they correlated to EGFR mutations could help to further clarify the importance of EGFR mutations on pathogenesis of NSCLC. In addition, we report the detection procedure of EGFR mutation using polymerase chain reaction-single strand conformational polymorphism (PCR–SSCP) method (Orita *et al*, 1989). As PCR–SSCP method is very suitable for detecting mutations within a relatively limited region such as EGFR gene mutations and has been already used to detect p53 gene mutations commercially, the method can be more easily applied to detect EGFR mutations in clinical setting than direct sequence.

## MATERIALS AND METHODS

### Patients and data collection

A total of 154 consecutive patients with NSCLC who underwent resection at the Department of Thoracic Surgery, Kyoto University Hospital, from January 2003 to November 2004 were included in the present study (Table 1). Clinical data of patients involved were obtained from the inpatient and outpatient medical records, chest X-ray films, whole-body computed tomography films, bone scanning data, and operation records. On smoking status, we classified patients into nonsmokers and smokers, and subclassified smokers into former smoker (who stop smoking at least 6 months before the time diagnosis of NSCLC) and current smoker. In total, 142 patients underwent complete lobe or segment in which the tumour existed and also received hilar and mediastinal lymphnode dissections. A total of 13 patients received partial lung resection and lymphnode sampling. No patient was exposed to gefitinib before his or her tumour was resected. Pathological staging was determined using the current tumour-node-metastasis classification system (UICC, 1997). The histological type and differentiation grade of the tumours in these patients were determined using the. Pathological diagnosis were performed by two pathologists, unaware of the genetic information, of Kyoto University Hospital Laboratory of Anatomic Pathology, and finally confirmed by one pathologist (TM) according to the WHO classification system (Travis *et al*, 1999). Many of adenocarcinomas were classified into mixed subtype according to WHO classification system. Thus, to clarify the impact of EGFR gene mutations on subtypes of adenocarcinoma, classification according to the presence or absence of each component (BAC, papillary, acinar, and solid carcinoma with mucin (solid)) in a tumour was also employed in this study. A written informed consent to perform genetic analyses was obtained from all patients before surgery, and the study itself was approved by the Ethics Committee of Kyoto University Graduate School and Faculty of Medicine.

### Tumour sample collection

Tumour tissues were frozen immediately after resection, and were stored at −80°C until DNA extraction. A part of each tumour tissue was used for formalin-fixed, paraffin-embedded tissue block to confirm that tumour cells were sufficiently included within the sample. Genomic DNA was extracted using the FastDNA® Kit (Qbiogene Inc., USA) as recommended by the manufacturer. As EGFR mutations were reported to be somatic, corresponding nonmalignant lung tissues were analysed in only six cases harbouring EGFR mutations in their tumour tissues, and all had no EGFR mutations in their nonmalignant lung tissues.

### Mutation detection and nucleotide sequence analysis of the EGFR and p53 genes

Polymerase chain reaction-single strand conformational polymorphism (Orita *et al*, 1989) was used to screen for mutations in the EGFR gene within exons 18–21 and for mutations in the p53 gene within exons 5–8. Polymerase chain reaction amplification was performed using the HotStarTaq Master Mix (Qiagen, Germany); the primers used and PCR conditions are listed in Table 2. Single-strand conformational polymorphism analyses were performed using the GenePhor System and GeneGel Excel 12.5/24 (Amersham Biosciences, Sweden) following the manufacturer's protocol; the gel temperature was maintained at 10°C for SSCP analysis of exon 21 of the EGFR gene and exon 6 of the p53 gene, 15°C for exons 18, 19, and 20 of the EGFR gene, and 18°C for exons 5, 7, and 8 of the p53 gene. After the gels were stained with silver carbonate, altered bands were cut from the gels and DNA fragments were eluted for direct sequencing. Each mutation within exon 19 of the EGFR gene was expediently named according to the start point of the amino-acid change and its order of detection, as shown in Table 3.

### Detection of mutations in codon 12 of the K-ras gene

We used a modified mutagenic PCR–RFLP method (Hatzaki *et al*, 2001) for screening mutations in codon 12 of the K-ras gene. PCR primers and the amplification conditions are shown in Table 2. The PCR products of mutated K-ras genes were sequenced for confirmation of mutation.

### Promoter hypermethylation analysis

To detect promoter hypermethylation of the p16, RASSF1A, and APC1A genes, methylation-specific PCR method was used (Herman *et al*, 1996). PCR primers and the amplification conditions were listed in Table 2 (Herman *et al*, 1996; Burbee *et al*, 2001; Yu *et al*, 2002).

### Statistical analyses

The significance of differences in categorical data was tested using the *χ*^2^ or Fisher's exact test. Differences between continuous variables were examined using the Mann–Whitney *U*-test. To determine which of gender or smoking history, or which component of subtype of adenocarcinoma affected EGFR mutations, logistic regression analyses were performed. StatView software (version 5, SAS Institute, USA) was used to carry out all statistical calculations. All statistical tests were two-sided, and differences were considered to be statistically significant if the *P*-value was less than 0.05.

## RESULTS

### Epidermal growth factor receptor mutations in non-small-cell lung carcinomas

**Figure 1:**
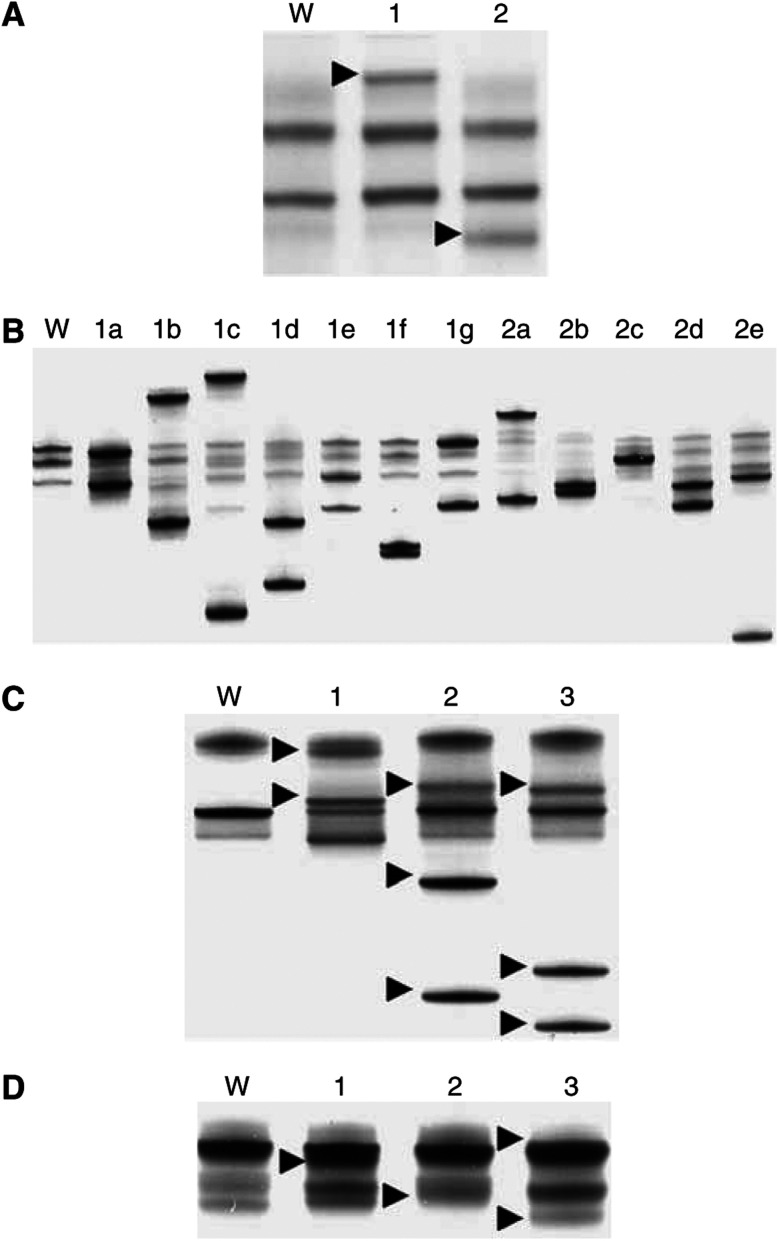
Single-strand conformation polymorphism of the EGFR gene. Each band alteration corresponds to a specific gene mutation. (**A**) Exon 18. W: wild type, 1: 2156G>C, 2: 2159C>T. Allow heads: altered bands. (**B**) Exon 19. W: wild type. The designated types of mutation (1a–1g, 2a–2e) in exon 19 were those described in Table 3. (**C**) Exon 20. W: wild type, 1: 2361G>A and 2407C>A, 2: 2308ins/dup(CCAGCGTGG) with 2310C>T and 2315C>G, 3: 2311ins/dup(GCGTGGACA) with 2315C>G. Allow heads: altered bands. (**D**) Exon 21. W: wild type, 1: 2573T>G, 2: 2573–2573CT>AG, 3: 2573–2574TG>GT. Allow heads: altered bands.

A total of 61 mutations in the EGFR gene were found in 60 of our patients (39.0%). Mutations occurred within exon 18 in two patients (1.3%), exon 19 in 34 patients (21.9%), exon 20 in three patients (1.9%), and exon 21 in 22 patients (14.2%), respectively. One patient had mutations within exon 19 and 20. Polymerase chain reaction-single strand conformational polymorphism analysis revealed two types of altered bands in exon 18 (Figure 1A), 12 types in exon 19 (Figure 1B), three types in exon 20 (Figure 1C), and three types in exon 21 (Figure 1D), and nucleotide sequencing confirmed the presence of the corresponding mutations shown in Table 3. The mutations identified within exon 18 were point mutation of 2156G>C (*n*=1), which substituted alanine for glycine at codon 719, and point mutation of 2159C>T (*n*=1), which substituted phenylalanine for serine at codon 720 (Table 3). A total of 12 types of mutations were discovered around codon 747–750 within exon 19, and 2235–2249del (*n*=14) and 2236–2250del (*n*=8) were major types (Table 3). The mutations identified within exon 20 were point mutation of 2361G>A and 2407C>A (silent mutation) observed in one patient who had deletion mutation within exon 19, and two types of duplication/insertion with point mutation (Table 3). The mutations identified within exon 21 were point mutation of 2573T>G (*n*=20), 2572–2573CT>AG (*n*=1), and 2573–2574TG>GT (*n*=1). All these mutations provided amino-acid substitution of arginine in the place of leucine at codon 858 (Table 3). Epidermal growth factor receptor mutations were exclusively observed in adenocarcinoma patients; the incidence of EGFR mutations in adenocarcinoma patients was 55.6% (60/108).

### Other genetic alterations

In total, 52 mutations within exons 5–8 of the p53 gene were observed in 51 patients (33.1%). The frequency distribution of these mutations was as follows: 12 missense point mutations and one duplication/insertion within exon 5; seven missense point mutations, two deletions, and two duplication/insertion within exon 6; four missense point mutations, three duplication/insertion, and two deletions within exon 7; and 15 missense point mutations, and four deletions within exon 8.

Mutations within codon 12 of the K-ras gene were observed in 10 patients (6.5%) with the following frequency: substitution of cystein (TGT) in the place of glycine (*n*=4), aspartic acid (GAT) (*n*=3), serine (AGT) (*n*=1), valine (GTT) (*n*=1), and phenylalanine (TTT) (*n*=1). All 10 patients with K-Ras mutated tumours were smokers.

Promoter hypermethylation of p16, RASSF1A, or APC1A genes was observed in 64 (41.6%), 78 (50.6%), or 82 (53.2%) of 154 patients, respectively.

### Correlation of clinico-pathological characteristics and other genetic alterations with EGFR mutations in lung adenocarcinoma

As EGFR mutations were observed exclusively in adenocarcinoma patients, we investigated the relationship between the clinical features and other genetic alterations in these adenocarcinoma patients (*n*=108) and their mutations (Table 4). The incidence of EGFR mutations was higher in female patients than in male patients (76.3 *vs* 34.0%, *P*<0.001, odds ratio: 6.3, 95% confidence intervals: 2.7–14.7). The incidence of EGFR mutations was higher in nonsmokers than in former smokers (83.0 *vs* 50.0%, *P*=0.008, odds ratio: 4.9, 95% confidence intervals: 1.5–15.7), and higher in former smokers than in current smokers (50.0 *vs* 15.2%, *P*=0.007, odds ratio: 5.6, 95% confidence intervals: 1.4–21.7). There was a significant correlation between gender and smoking status in these patients; 48 of 53 male patients (90.6%) were smokers, whereas only seven of 55 female patients (12.7%) were smokers (*P*<0.001, odds ratio: 65.8, 95% confidence intervals: 23.3–186.1). Logistic regression analysis revealed absence of smoking history, not female, affected EGFR gene mutation (*P*<0.001, odds ratio: 11.4, 95% confidence intervals: 2.7–47.3).

Epidermal growth factor receptor mutations were more frequently found in patients with lower pathologic stage disease although there was no statistical significance (*P*=0.059) (Table 4).

The histological subtype of adenocarcinomas according to WHO classification did not correlate statistically with their EGFR incidence of mutations. Epidermal growth factor receptor mutations were, however, more frequently observed in tumours with BAC component than those without BAC component (78.9 *vs* 42.9%, *P*<0.001, odds ratio: 5.0, 95% confidence intervals: 2.0–12.6). By contraries, EGFR mutations were less frequently observed in tumours with solid component than those without solid component (34.2 *vs* 67.1%, *P*=0.001, odds ratio: 0.25, 95% confidence intervals: 0.11–0.61). Papillary component and acinar component did not correlate to the incidence of EGFR gene mutations (Table 4). Logistic regression analysis revealed that BAC component positively related to EGFR gene mutations (*P*=0.006, odds ratio: 3.9, 95% confidence intervals: 1.5–10.1) and solid component inversely related to EGFR gene mutations (*P*=0.035, odds ratio: 0.36, 95% confidence intervals: 0.14–0.93). The differentiation grade of the tumours correlated significantly with their incidence of EGFR mutations. Thus, incidence of EGFR mutations was lower in poorly differentiated tumours than in well-differentiated tumours (34.2 *vs* 72.0%, *P*=0.005, odds ratio: 4.9, 95% confidence intervals: 1.5–15.9) or than in moderately differentiated tumours (34.2 *vs* 64.4%, *P*=0.008, odds ratio: 3.5, 95% confidence intervals: 1.3–9.2) (Table 4).

K-ras gene mutations were not detected in any of the EGFR mutated tumours and this negative correlation was statistically significant (*P*=0.001), while EGFR gene mutation status did not correlate with p53 gene mutation status or with promoter hypermethylation status of p16, RASSF1A, or APC1A gene (Table 4).

The detailed type of EGFR mutation did not correlate with gender, smoking status, p-stage, histological subtype, grade of tumour differentiation (data not shown).

## DISCUSSION

In the present study, we detected EGFR gene mutations in 60 of 154 Japanese patients (39.0%) who underwent resection for NSCLC. The demonstration of a high prevalence of these mutations in our Japanese patients was consistent with previous data that NSCLC occurred in people in the East Asia including Japan showed higher prevalence of EGFR mutation (19–40%) than those in other patient groups (4–10%) (Table 5). Our study clearly showed that EGFR mutations were not only observed in advanced NSCLC that may be considered for gefitinib treatment but also in early NSCLC. This suggests that such mutations is involved in the early stage of oncogenesis of NSCLC, so that EGFR mutations should be investigated further in regard to oncogenesis of NSCLC, as well as considered for design of clinical trial of or selecting candidates of EGFR-targeting drugs.

A total of 15 base pair deletions within exon 19 and a point mutation within exon 21 were two of the major types of mutations that were found in our patients. Among 11 types of EGFR mutations within exon 19, mutations of the type 1 series, in which the start point for amino acid deletion was E746, were most frequent. This finding was somewhat different from the findings in US patients, in whom mutations that had their start point for amino acid deletion at L747 were frequent (Lynch *et al*, 2004; Paez *et al*, 2004), but was similar to those in other reports (Huang *et al*, 2004; Kosaka *et al*, 2004; Pao *et al*, 2004; Han *et al*, 2005; Marchetti *et al*, 2005; Qin *et al*, 2005; Shigematsu *et al*, 2005; Yang *et al*, 2005). These differences may reflect ethnic and/or social differences among patient groups although the net effect of a deletion in the type 1 or 2 series on sensitivity to EGFR tyrosine kinase inhibitors seems similar (Huang *et al*, 2004; Lynch *et al*, 2004; Paez *et al*, 2004; Pao *et al*, 2004; Han *et al*, 2005).

It is interesting that both mutations in exons 19 and 21 were found with higher frequency (34/154 and 22/154, respectively) in our patients than in patients outside of the East Asia (Table 5), despite the type of each mutation were different (a deletion on exon 19 and a point mutation on exon 21). This finding suggests that one or just a few substances with DNA editing capacity may mediate both deletions and point mutations within the tyrosine kinase domain of the EGFR. Clarification of the mechanism by which EGFR mutations occur can lead to advances in our understanding of oncogenesis and its prevention.

Smoking history strongly affected the incidence of EGFR mutations in our study. In addition to higher incidence of EGFR mutations in adenocarcinomas developed in nonsmokers, we showed that the incidence of EGFR mutations was higher in former smokers than in current smokers. Former smokers had lower pack-year (Table 1) and may be less affected by smoking. Probably smoking is not involved in mechanisms of EGFR gene mutations.

Although the incidences of EGFR mutations were higher in female patients and in nonsmokers, a history of smoking strongly correlated with gender in our study. We showed that the absence of smoking history, but not female, independently affected EGFR gene mutations. This result is similar to the data of Kosaka *et al* (2004), but is different from the data of Marchetti *et al*, which indicated the absence of smoking history and female sex independently influenced to EGFR mutations. This difference may be derived from social difference on smoking between Japan and Italy. At least, in NSCLC occurred in Japanese people, the absence of smoking history, not female gender, seems a critical factor that links to the prevalence of EGFR mutations.

We found that BAC component positively related to EGFR mutations, solid component inversely related to EGFR gene mutations, and that well-to-moderately differentiated adenocarcinomas had higher incidence of EGFR gene mutations than poorly differentiated adenocarcinomas. These findings are consistent with Lynch *et al* (2004), Kosaka *et al* (2004), and Marchetti *et al* (2005). Adenocarcinomas that develop in nonsmokers frequently display features of BAC and papillary type tumours, whereas those that develop in smokers frequently include poorly differentiated and solid subtype tumours (Hashimoto *et al*, 2000; Yang *et al*, 2002; Nordquist *et al*, 2004). High incidence of harbouring EGFR mutations in nonsmokers adenocarcinoma well-explains this predilection on pathological findings. Thus, including BAC component and well-to-moderate differentiation grade seem histological features of adenocarcinomas with EGFR mutations.

K-ras mutations were not observed in any of the EGFR-mutated tumours in our patients. This feature is similar to reports referring EGFR mutations and K-ras mutations (Table 5). Both K-ras and EGFR are important molecules that are responsible to the regulation of the mitogen-activated protein kinase pathway. But K-ras mutations are linked to the development of adenocarcinomas in smokers (Ahrendt *et al*, 2001), and are rarely observed in adenocarcinomas in nonsmokers (Noda *et al*, 2001). This is in marked contrast to EGFR mutations, which occur more frequently in nonsmokers. Probably, EGFR mutations have a similar significance to K-ras mutations in oncogenesis of lung adenocarcinomas. Unlike K-ras mutations involved in adenocarcinomas developed in smokers, however, EGFR gene mutations may play a key role in the development of adenocarcinomas in nonsmokers.

Similar to Kosaka *et al* (2004), EGFR mutations did not correlate to p53 gene mutations in our study. Moreover, we showed that EGFR mutations did not correlate to promoter hypermethylation status of p16, RASSF1A, or APC1A genes. In the multistep progression of sporadic colorectal carcinomas, K-ras mutations are thought to occur independently at a different step from that of p53 mutations (Klump *et al*, 2004). Similar situation is seen in K-ras mutations, p53 mutations, and promoter hypermethylation of p16 gene in pancreatic cancer (Moore *et al*, 2003). In NSCLC, EGFR, p53, p16, RASSF1A, or APC1A can be involved in oncogenesis at a different level from one another (Rom *et al*, 2000). Therefore, alterations of these genes can independently occur in lung adenocarcinomas, unlike mutations of EGFR and K-ras.

We used the PCR–SSCP method (Orita *et al*, 1989) to screen for EGFR mutations. Advantages of the PCR–SSCP method are: it is fast and easily employed for screening numerous samples simultaneously, it indicates type-specific mutations without nucleotide sequence because one altered SSCP-band pattern can correspond to a specific mutation, and it has higher sensitivity than direct sequence sufficient for clinical use (Marchetti *et al*, 2005). The PCR–SSCP method can be theoretically applied not only to resected tumour samples but also to sputum, pleural effusion, and biopsy specimens; as such, this technique can be used to preselect appropriate patients for EGFR tyrosine kinase inhibitor treatment.

This study has several limitations. As only surgically resected tumours were involved in the study, the incidence of EGFR mutations in the study could not indicate the incidence in whole NSCLCs. Mutations outside exons 18–21 were not examined, and PCR–SSCP method has possibility to overlook mutations that is not reflected on band pattern alterations, so that the incidence of EGFR may be underestimated. The incidence of EGFR mutation and types of mutations detected in our study are, however, quite similar to previous reports; therefore, we think our study has sufficient validity.

In conclusion, mutations in the EGFR gene were found in approximately half of our Japanese adenocarcinoma patients, and were more common in tumours developed in nonsmokers. Adenocarcinomas with EGFR mutations displayed inclusion of bronchio-alveolar component, or well-to-moderately differentiated features, which are also one of the histological features of adenocarcinomas in nonsmokers. Adenocarcinomas with EGFR mutations negatively correlated with K-ras mutations that are known to be associated with smoking. Thus, EGFR mutations may play a role in the aetiology of adenocarcinoma in nonsmokers.

## Figures and Tables

**Table 1:** Characteristics of 154 patients included in the study

**Patients characteristics**	
*Age (year)*	
Median (range)	68 (31–83)

*Gender (no.)*	
Female (%)	60 (39.0%)
Male (%)	94 (61.0%)

*Smoking status (no.)*	
Non smoker (%)	56 (36.4%)
Smoker (%)	98 (63.6%)
Former	29
Current	69

*Pack-year of smokers (pack-year)*	
All smokers	
Median (range)	48 (2–250)
Former	
Median (range)	30 (2–105)
Current	
Median (range)	51 (6–250)

*Tumour histology (no.)*	
Adenocarcinoma	108 (70.1%)
Squamous cell	31 (20.1%)
Large cell	9 (5.9%)
Other histologies	6 (3.9%)

*Pathological stage (no.)*	
IA	63 (41.3%)
IB	38 (24.5%)
IIA	4 (2.6%)
IIB	11 (7.1%)
IIIA	26 (16.8%)
IIIB	10 (6.4%)
IV	2 (1.3%)

**Table 2:** PCR primers and parameters

**Gene**	**Forward (5′–3′)**	**Reverse (5′–3′)**	**Product size (bp)**	**Number of cycle**	**Annealing condition**
*EGFR*					
Exon 18	TACACCCAGTGGAGAAGCTCC	CCCCACCAGACCATGAGAG	169	30	58°C, 30 s
Exon 19	CAATTGCCAGTTAACGTCTTCC	GGAGATGAGCAGGGTCTAGAG	239	30	58°C, 30 s
Exon 20	CACACTGACGTGCCTCTC	CTTATCTCCCCTCCCCGTA	252	30	56°C, 30 s
Exon 21	AGGGCATGAACTACTTG	CCTCCTTACTTTGCCTCCTTC	167	35	55°C, 30 s

*p53*					
Exon 5	TTCAACTCTGTCTCCTTCCT	CAGCCCTGTCGTCTCTCCAG	248	30	55°C, 30 s
Exon 6	GCCTCTGATTCCTCACTGAT	TTAACCCCTCCTCCCAGAGA	181	30	55°C, 30 s
Exon 7	CTTGCCACAGGTCTCCCCAA	TGTGCAGGGTGGCAAGTGGC	196	30	59°C, 30 s
Exon 8	TTCCTTACTGCCTCTTGCTT	CGCTTCTTGTCCTGCTTGCT	201	30	55°C, 30 s

K-ras	ACTGAATATAAACTTGTGGTAGTTGGACCT	CTGTATCAAAGAATGGTCCTGCACCAGTA	162	30	58°C, 30 s

*p16*					
Unmethylated	TTATTAGAGGGTGGGGTGGATTGT	CAACCCCAAACCACAACCATAA	151	35	60°C, 30 s
Methylated	TTATTAGAGGGTGGGGCGGATCGC	GACCCCGAACCGCGACCGTAA	150	35	65°C, 15 s

*RASSF1A*					
Methylated	GGGTTTTGCGAGAGCGCG	GCTAACAAACGCGAACCG	169	35	64°C, 50 s

*APC1A*					
Methylated	TATTGCGGAGTGCGGGTC	TCGACGAACTCCCGACGA	98	35	62°C, 10 s

**Table 3:** Types of EGFR gene mutations found in this study

**Exon**	**Type of sequence**	**Alteration**	**Nucleotide alteration**	**Amino-acid alteration**	**No. of cases**
18		Substitution	2156G>C	G719A	1
		Substitution	2159C>T	S720F	1

19	*Type 1*				
	1a	Deletion	2235–2249delGGAATTAAGAGAAGC	E746-A750del	14
	1b	Deletion	2236–2250delGAATTAAGAGAAGCA	E746-A750del	8
	1c	Deletion+	2235–2249delGGAATTAAGAGAAGC	E746-A750del	1
		Substitution	2251A>G	T751A	
	1d	Deletion+	2235–2236delGC	E746-A750del	1
		Substitution	2242–2248delAGAGAAG	ins I and P	
			2241A>C		
	1e	Deletion	2235–2236delGC	E746-T751del	2
			2239–2252delTAAGAGAAGCAAC	ins I	
	1f	Deletion+	2235–2236delGC	E746-T751del	1
		Substitution	2242–2251delAGAGAAGCAA	ins I and P	
			2241A>C		
	1g	Deletion+	2237–2254delAATTAAGAGAAGCAACAT	E746-S752del	1
		Substitution	2255C>T	ins V	
	*Type 2*				
	2a	Deletion+	2240–2248delTAAGAGAAG	L747-A750	1
		Substitution	2239T>C	insP	
	2b	Deletion+	2240–2251delTAAGAGAAGCAA	L747-T751del	2
		Substitution	2239T>C	ins S	
	2c	Deletion	2239–2253delTTAAGAGAAGCAACA	L747-T751del	1
	2d	Deletion	2239–2256delTTAAGAGAAGCAACATCT	L747-S752del	1
	2e	Deletion	2240–2257delTAAGAGAAGCAACATCTC	L747-P753del	1
				ins S	

20		Insertion+	2308ins/dup(CCAGCGTGG)	ins779(ASV)	1
		Substitution	2310C>T, 2315C>G	P782R	
		Insertion+	2311ins/dup(GCGTGGACA)	ins780(SVD)	1
		Substitution	2315C>G	P782R	

21		Substitution	2573T>G	L858R	20
		Substitution	2572–2573CT>AG	L858R	1
		Substitution	2573–2574TG>GT	L858R	1

**Table 4:**
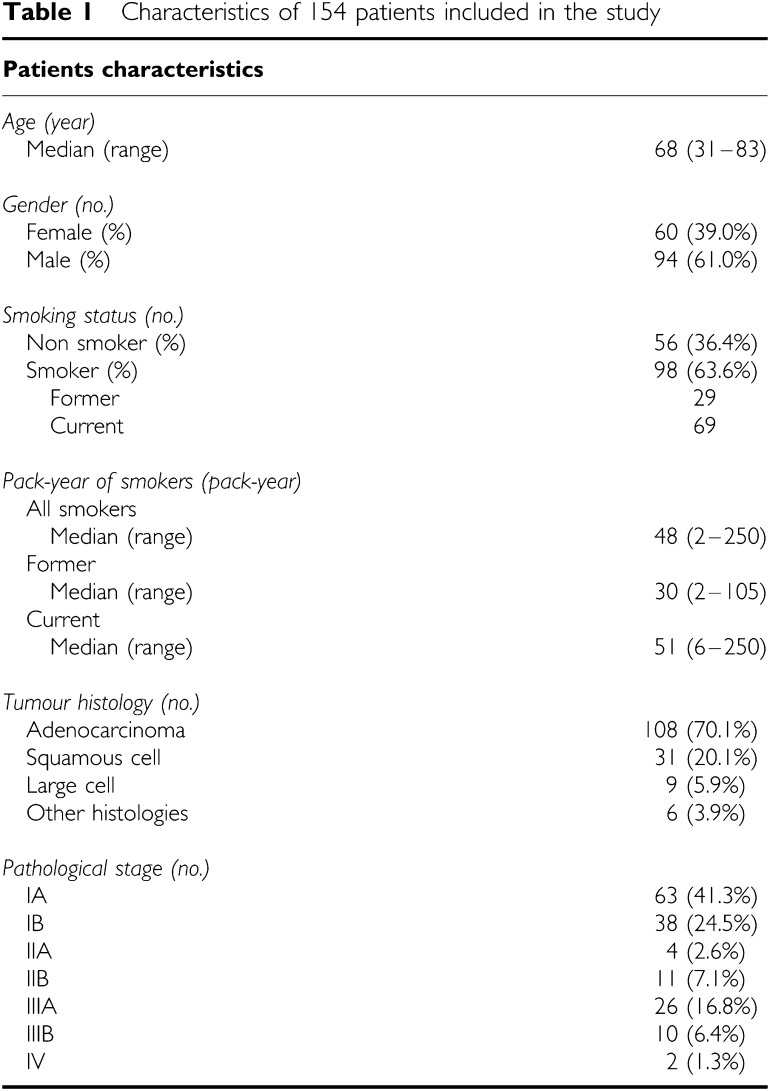
Relationship of EGFR gene mutations to clinicopathological characteristics and other genetic/epigenetic alterations in 108 patients with adenocarcinoma

**Table 5:** Summary of previous reports on EGFR gene mutations in NSCLC

**Reference**	**Patient group**	**No. of patients**	**Frequency in**		**Predilection in non/former smokers**	**Histological features in Ad^a^**	**Respose to TKI^b^of patients with EGFR mutated tumour**	**Correlation to p53**	**Correlation to K-ras**
			**Overall**	**Ad^a^**					
Lynch *et al* (2004)	USA	25 +16 TKI^b^ receivers	2/25	2/22	Yes	More frequent in BAC^c^	8 patients in 9 responders not in 7 nonresponders	—	—

Paez *et al* (2004)	USA Japan	119 +9 TKI^b^ receivers	16/119 (13%) 1/61(2%) in USA 15/58 (26%) in Japan	15/70 (21%) 1/29(3%) in USA 14/41(34%) in Japan	Yes	—	5 patients in 5 responders not in nonresponders	—	—

Pao *et al* (2004)	USA	96 +17 TKI b responders	11/96 (11%)	—	Yes	—	12 patients in 17 responders	—	—

Kosaka *et al* (2004)	Japan	277	111/277 (40%)	110/224 (49%)	Yes	More frequent in well-to-mederately differentiated Ad^a^	—	Independent	Mutually exclusive

Huang *et al* (2004)	Taiwan	101	39/101 (39%)	38/69 (55%)	Yes	—	7 responders in 9 patients	—	—

Marchetti *et al* (2005)	Italy	860	37/860 (4%)	37/375 (10%)	Yes	More frequent in Ad with BAC^c^ features	—	—	Mutually exclusive

Han *et al* (2005)	Korea	90	17/90 (19%)	14/65 (22%)	No	—	11 responders in 17 patients	—	—

Shigematsu *et al* (2005)	Japan Taiwan USA Australia	519	120/519 (23%) 107/361 (30%) in East Asia 13/158(8%) in others	114/289 (39%) 102/214 (48%) in East Asia 12/75(16%) in others	Yes	No correlation to Ad with BAC^c^ fearures	—	—	Mutually exclusive

Qin *et al* (2005)	China	41	10/41 (24%)	7/17 (41%)	No	—	—	—	—

Yang *et al* (2002)	USA	219	26/219 (12%)	25/164 (15%)	Yes	—	—	—	—

a Ad=adenocarcinoma.

b TKI=tyrosine kinase inhibitor.

c BAC=bronchio-alveolar carcinoma.
